# Dipeptidyl Peptidase-4 Inhibitors and the Risk of Pancreatitis in Patients with Type 2 Diabetes Mellitus: A Population-Based Cohort Study

**DOI:** 10.1155/2018/5246976

**Published:** 2018-04-10

**Authors:** Young-Gun Kim, Seirhan Kim, Seung Jin Han, Dae Jung Kim, Kwan-Woo Lee, Hae Jin Kim

**Affiliations:** ^1^Department of Medical Sciences, Ajou University Graduate School, Suwon, Republic of Korea; ^2^Department of Internal Medicine, Incheon Medical Center, Incheon, Republic of Korea; ^3^Korean Centers for Disease Control and Prevention, Ministry of Health and Welfare, Seoul, Republic of Korea; ^4^Department of Endocrinology and Metabolism, Ajou University School of Medicine, Suwon, Republic of Korea

## Abstract

**Background:**

Information on the risk of acute pancreatitis in patients receiving dipeptidyl-peptidase IV inhibitors (DPP-4i) is limited and controversial. One study suggested that the differences in findings between these meta-analyses were attributed to whether they included large randomized control trials with cardiovascular outcomes or not. The aim of our study was to determine whether the use of DPP-4i increases the risk of acute pancreatitis compared with sulfonylurea (SU) and whether the risk is higher in patients with underlying cardiovascular disease (CVD).

**Methods:**

A population-based cohort study was performed using Korean National Health Insurance Service-National Sample Cohort data. We included 33,395 new users of SU and DPP-4i from 1 January 2008 to 31 December 2015. SU-treated patients and DPP-4i-treated patients were matched by 1 : 1 propensity score matching. We used Kaplan–Meier curves and Cox proportional hazards regression analysis to calculate the risk of acute pancreatitis.

**Results:**

The hazard ratio (HR) of hospitalization for acute pancreatitis was 0.642 (95% confidence interval (CI): 0.535–0.771) in DPP-4i-treated patients compared with SU-treated patients. The HR of DPP-4i use was also lower than that of SU use in patients without underlying CVD (HR: 0.591; 95% CI: 0.476–0.735) but not in patients with underlying CVD (HR: 0.727; 95% CI: 0.527–1.003).

**Conclusion:**

Our findings suggest that DPP-4i is less likely to cause drug-induced pancreatitis than SU. This finding was not evident in patients with CVD, but DPP-4i was not more likely to induce pancreatitis in these patients than SU was.

## 1. Introduction

Dipeptidyl-peptidase IV inhibitors (DPP-4i) are widely prescribed for the treatment of type 2 diabetes mellitus (T2DM) because of their several advantages; they effectively control blood sugar, pose a low risk of hypoglycemia, and are neutral for weight [1]. Since the initial release of DPP-4i, more evidence on the safety of DPP-4i has accumulated. The United States Food and Drug Administration Adverse Event Reporting System has reported cases of acute pancreatitis that were likely provoked by DPP-4i use, including necrotizing or hemorrhagic pancreatitis, which can be life threatening [2]. Acute pancreatitis is a serious disease that causes severe abdominal pain and dyspepsia and leads to hospital admission. Furthermore, acute pancreatitis can cause another acute pancreatitis or chronic pancreatitis in 10–20% of patients [3]. Due to increasing prescription of DPP-4i and the clinical significance of pancreatitis, there is a growing interest in the risk of pancreatitis from DPP-4i.

Several observational and meta-analysis studies have been conducted. However, these studies had conflicting results. Three observational studies that compared DPP-4i users with nonusers concluded that DPP-4i did not increase the risk of pancreatitis [4–6]. However, in another study, DPP-4i users showed an increased risk of pancreatitis compared with nonusers [7]. The differences in these results may be explained by the different proportion of oral hypoglycemic agent (OHA) use in the control group, as some OHAs such as sulfonylurea (SU) and metformin are reported risk factors for pancreatitis [8–10]. Moreover, since no clear clinical information was obtained from these studies in terms of comparisons of DPP-4i agents with other OHAs, studies comparing the risk of pancreatitis between DPP-4i and specific OHAs may be more clinically informative. Among many oral hypoglycemic agents, SU is one of the most frequently used second-line agent add-ons to metformin and acts on insulin secretion, similar to DPP-4i. Therefore, studies comparing the risk of DPP-4i pancreatitis with SU are needed.

The results of several meta-analyses were also controversial. Some studies showed that DPP-4i did not increase the risk of acute pancreatitis [11, 12], while others concluded that it did [13–16]. One study suggested that the different results from those studies was related to whether they included the following three particular large randomized control trials (RCTs) evaluating cardiovascular outcomes: the Saxagliptin Assessment of Vascular Outcomes Recorded in Patients with Diabetes Mellitus-Thrombolysis in Myocardial Infarction 53 (SAVOR-TIMI 53), Examination of Cardiovascular Outcomes with Alogliptin versus Standard of Care (EXAMINE) and Trial Evaluating Cardiovascular Outcomes with Sitagliptin (TECOS) [17]. They suggested that DPP-4i increases the risk of pancreatitis in patients with underlying cardiovascular disease (CVD). However, no study has examined whether DPP-4i is likely to cause pancreatitis in patients with underlying CVD.

We sought to evaluate the acute pancreatitis risk of DPP-4i compared with SU in a population-based cohort study using a national health insurance database. We also assessed whether the risk of pancreatitis from DPP-4i is influenced by the presence of underlying CVD.

## 2. Methods

### 2.1. Study Design and Data

In South Korea, the Korean National Health Insurance Service covers over 99% of the population, and its database contains comprehensive medical information, including claims related to drug prescriptions, procedures, diagnoses, and patient demographics. We conducted a population-based retrospective observational cohort study using the database of the Korean National Health Insurance Service-National Sample Cohort, involving almost 1,000,000 people and their claims data. This database obtained a solid representation of the Korean population by selecting patients using a stratified random sampling method with 1476 strata from 1 January 2002 to 31 December 2015 [18]. The diagnoses were coded using the International Classification of Diseases, 10th revision. This study was performed with the approval of the Institutional Review Board (IRB) of Ajou University Hospital. Informed consent was waived by the IRB.

### 2.2. Study Population and Follow-Up Period

**Figure 1:**
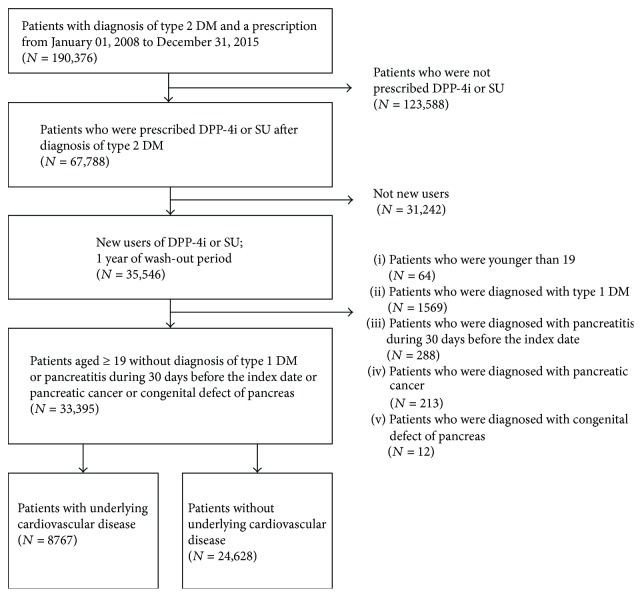
Flow chart of the sample selection, stratified by underlying cardiovascular disease. DM: diabetes mellitus; DPP-4i: dipeptidyl-peptidase IV inhibitor; *N*: number of patients; SU: sulfonylurea.

From 1 January 2008 to 31 December 2015, new users older than 19 years who were diagnosed with type 2 DM and prescribed DPP-4i or SU were enrolled in the cohort. A 1-year washout period was used to identify new users of DPP-4i or SU. The date of the first prescription for DPP-4i or SU was designated as the index date, and the prescribed drug was defined as the index drug. Patients who had been diagnosed with type 1 DM, pancreatic cancer, or congenital defects of the pancreas were excluded. Patients who had been diagnosed with pancreatitis within 30 days before their index date were excluded. The patient selection is presented in Figure 1.

We stratified patients according to CVD to determine if DPP-4i increased the risk of pancreatitis in these patients. Patients who had been diagnosed with myocardial infarction, unstable angina, other ischemic heart disease, cerebral infarction, other cerebrovascular event, or peripheral arterial occlusive disease were classified as those with underlying CVD.

The follow-up period was defined from the index date to either the date of index drug discontinuance, >30-day gap between index drug prescriptions, prescription of the opposite drug, or the study end date (31 December 2015), whichever occurred first.

### 2.3. Study Outcome and Subgroup Analysis

The study outcome was hospitalization for acute pancreatitis. Time to outcome was defined as the number of days from the index date to the first occurrence of the outcome event. Subgroup analyses were performed according to sex, age (<65 and ≥65 years), and the presence of DM microvascular complications. Patients who had been diagnosed with DM nephropathy, neuropathy, or retinopathy were defined as patients with underlying DM microvascular complications.

### 2.4. Statistical Analysis

R software (ver. 3.3.3; R Development Core Team, Vienna, Austria) and SAS (ver. 9.4; SAS Institute, Cary, NC, USA) were used for the statistical analyses. Data were expressed as means ± standard deviation. To minimize various biases between patients prescribed SU and patients prescribed DPP-4i, we used propensity score matching. Among several propensity score matching methods, we used the nearest neighbor technique with a caliper of 0.01 on the probability scale, and replacement of the control was not permitted. Propensity scores were calculated using multivariate logistic regression analysis for all of the variables presented in Table 1: age, sex, diagnoses (1 year before the index date), prescribed drugs (180 days before the index date), and procedures (1 year before the index date). Propensity score matching was performed in the entire cohort and in each subgroup. The quality of correction of confounding variables between the two groups was evaluated as a standardized difference. An absolute value of the standardized difference < 0.1 was considered a negligible difference between groups.

Kaplan–Meier estimates and the Cox proportional hazards model were used to estimate the effect of DPP-4i on acute pancreatitis. For the Kaplan–Meier estimate, 1 minus the Kaplan–Meier estimate was used. Cox proportional hazards regression was first performed for all propensity score-matched patients and then for patients in each subgroup.

## 3. Results

**Figure 2:**
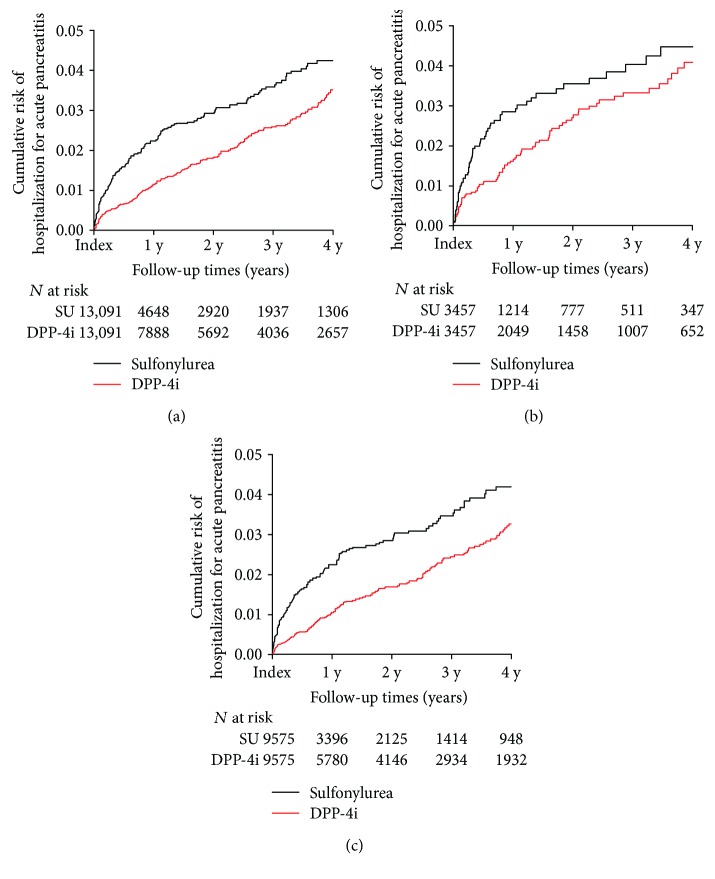
Kaplan–Meier plots of hospitalization for acute pancreatitis in (a) all patients, (b) patients with underlying cardiovascular disease, and (c) patients without underlying cardiovascular disease. DPP-4i: dipeptidyl-peptidase IV inhibitor; *N*: number of patients; SU: sulfonylurea; y: year(s).

The study cohort included 33,395 patients: 14,399 in the SU group and 18,996 in the DPP-4i group. In total, 56,953 person-years were considered. After propensity score matching, 13,091 patients were included in each group. The baseline characteristics of the matched group are presented in Table 1, and the standardized mean difference was 0.46% (SD = 0.43%, standardized differences of all variables were <0.1). The mean follow-up period of the matched patients was 627.6 days. During the follow-up period, 468 patients were hospitalized for acute pancreatitis. The hazard ratio (HR) for hospitalization for acute pancreatitis was lower in the DPP-4i-treated than in the SU-treated patients (HR: 0.642; 95% confidence interval (CI): 0.535–0.771; *P* < 0.001; Figure 2, Table 2).

Patients were stratified by the presence of underlying CVD. After propensity score matching, 3457 patients were included in each group with underlying CVD and 9575 patients in each group without underlying CVD. The standardized difference of all variables was <0.1, and the standardized mean difference was 0.84% (SD = 0.48%) in patients with CVD and 0.69% (SD = 0.71%) in patients without CVD. The HR for hospital admission for acute pancreatitis was 0.727 in DPP-4i-treated patients with CVD but was not significantly different from that in SU-treated patients (95% CI: 0.527–1.003; *P* = 0.052; Figure 2, Table 2). However, the HR for hospital admission was significantly lower in DPP-4i-treated than in SU-treated patients without CVD (HR: 0.591; CI: 0.476–0.735; *P* < 0.001; Figure 2, Table 2).

**Figure 3:**
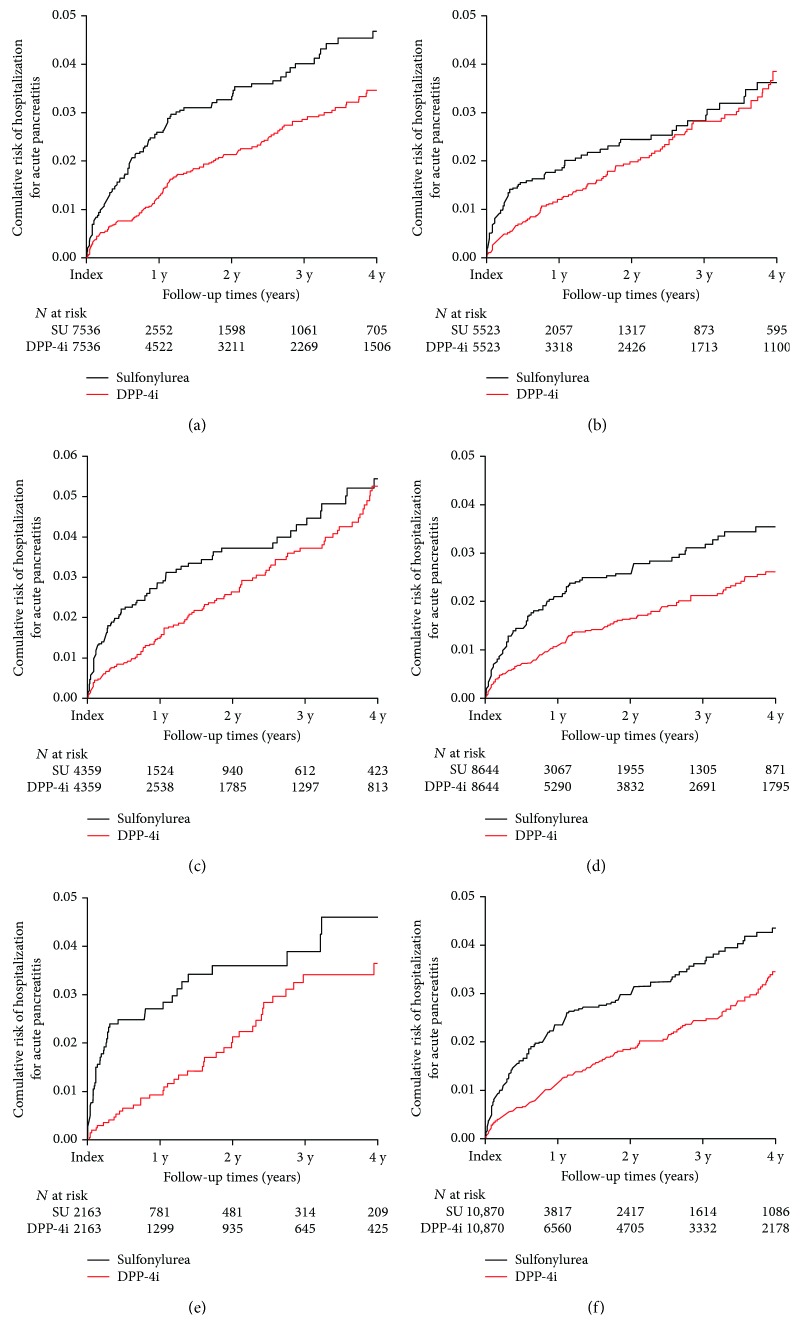
Kaplan–Meier plots for hospitalization for acute pancreatitis in male patients (a), female patients (b), patients aged ≥ 65 years (c), patients aged < 65 years (d), patients with DM microvascular complications (e), and patients without DM microvascular complications (f). DM: diabetes mellitus; DPP-4i: dipeptidyl-peptidase IV inhibitor; *N*: number of patients; SU: sulfonylurea; y: year(s).

In the subgroup analysis, patients were grouped according to sex, age (≥65 and <65 years), or presence of DM microvascular complications. Baseline characteristics of matched pairs in each subgroup are presented in Supplementary Tables 1, 2, and 3. DPP-4i showed protective effects on acute pancreatitis compared with SU in male patients (HR: 0.624; 95% CI: 0.495–0.788; *P* < 0.001; Figure 3(a), Table 2) but not in female patients (HR: 0.797; 95% CI: 0.600–1.058; *P* = 0.116; Figure 3(b), Table 2). DPP-4i use showed protective effects on acute pancreatitis compared with SU in both patients ≥ 65 years (HR: 0.717; 95%: CI 0.545–0.943; *P* = 0.017) and those < 65 years of age (HR: 0.617; 95% CI: 0.485–0.756; *P* < 0.001; Figures 3(c) and 3(d), Table 2). DPP-4i use showed a lower HR compared with SU use in both patients with (HR: 0.542; 95% CI: 0.357–0.822; *P* = 0.004) and without DM microvascular complications (HR: 0.622; 95% CI: 0.509–0.760; *P* < 0.001; Figures 3(e) and 3(f), Table 2).

## 4. Discussion

The results of our cohort study revealed that DPP-4i use is associated with a lower risk of hospitalization for acute pancreatitis compared with SU use. Compared with SU use, DPP-4i use showed a similar risk of hospitalization for acute pancreatitis in patients with T2DM and underlying CVD but a lower risk in patients without underlying CVD with statistical significance. In the subgroup analysis, DPP-4i use showed a lower HR compared with SU use regardless of age or presence of DM microvascular complications. This tendency was also observed in male patients, but not in female patients.

Two animal studies showed a correlation between DPP-4i use and acute pancreatitis. Sitagliptin-treated rats showed pancreatic ductal turnover, ductal metaplasia, and even pancreatitis [19, 20]. The suggested possible underlying mechanism was as follows: the glucagon-like peptide 1 receptor, which is abundantly expressed in the pancreatic duct and islets, is stimulated by DPP-4i via inhibition of DPP-4 and increased glucagon-like peptide 1 [21], resulting in overgrowth of pancreatic acinar and ductal cells into the small pancreatic ducts; this could lead to ductal occlusion, thereby triggering pancreatitis [22]. However, these two studies used very high doses of DPP-4i, which are not used in humans; therefore, these results are not reproducible across all studies and do not occur with all incretin-based therapies [19, 23, 24]. In another study, tissues from multiple animal species treated with sitagliptin did not show any evidence of pancreatitis [24]. Furthermore, in a recently published RCT, sitagliptin use in T2DM patients showed a “brief and modest increase of plasma pancreatic enzyme,” but “pancreatic exocrine function was unaffected” [25].

The results of observational studies on the risk of pancreatitis in DPP-4i are controversial. One retrospective observational study showed an increased risk of pancreatitis using the United Kingdom Clinical Practice Research Datalink (UK CPRD) database [7]. However, Azoulay et al. also analyzed the same UK CPRD data in combination with data from the United States and from five Canadian provinces and concluded that DPP-4i does not increase the risk of pancreatitis [4]. One possible reason for the discrepancy between the two studies is that both studies used DPP-4i nonusers as controls. Using DPP-4i nonusers as a control group when assessing the risk of pancreatitis in patients treated with DPP-4i could be a limitation for the studies, because some studies have reported that metformin and SU increase the risk of pancreatitis [8–10]. One systematic review showed that biguanide was reportedly associated with acute pancreatitis only in some case reports and SU was correlated with acute pancreatitis in two observational studies and two case reports [8].

Two observational studies compared the risk of acute pancreatitis between DPP-4i and SU use [26, 27]. One study showed that DPP-4i poses a similar risk of acute pancreatitis compared with SU using UK CPRD database [26], unlike our result that DPP-4i poses a lower risk. They adjusted confounding variables by calculating propensity scores and matching both groups, which was similar to our study design. However, the database they used contained claims data from only 680 general practices in the UK and medical histories such as the diagnosis of acute pancreatitis, admission history, and drug prescriptions from specialists were missing. In addition, while many observational studies have categorized hospitalizations for acute pancreatitis as outcomes [4–6, 9, 10, 28], this study only defined diagnosis of acute pancreatitis as an outcome. These two differences may have contributed to the different results between that study and ours. In line with our results, Chang et al. found that DPP-4i was protective over SU with regard to pancreatitis development, and they also used a national claims database that included health records from specialists [27].

Conflicting results have also been found in several meta-analyses conducted to date. One meta-analysis of 55 RCTs concluded that DPP-4i does not increase the risk of pancreatitis [11]. Another study, which included 69 RCTs, found that vildagliptin did not increase the risk of pancreatitis compared with a placebo [12]. However, other meta-analyses that included three large phase III RCTs (the SAVOR-TIMI 53, EXAMINE, and TECOS trials) evaluated the cardiovascular risk associated with DPP-4i and concluded that the use of DPP-4i increased acute pancreatitis compared with a placebo [13, 14]. Analysis of only those three RCTs resulted in a higher HR (1.78, 95% CI: 1.13–2.81) for the risk of pancreatitis [15, 16]. With regard to this discrepancy, Sohani et al. suggested that DPP-4i increases the risk of pancreatitis in patients with CVD [17], although the exact mechanism is not yet known. Although those meta-analyses cannot be directly compared with our study, because those meta-analyses included only placebo-controlled RCTs, our study showed that DPP-4i has a similar pancreatitis risk with SU in patients with CVD, unlike patients without CVD. DPP-4i use could pose a risk of pancreatitis in patients with CVD, but this risk is similar to that posed by SU.

In the subgroup analysis, DPP-4i use did not show a lower HR for pancreatitis than did SU use in female patients, unlike for the entire patient group or for male patients. Those differences may be due to lifestyle factors such as smoking or alcohol intake, body composition, sex hormones, or physical activity. In Korea, according to the National Health and Nutrition Examination Survey, the rates of alcohol consumption and smoking differ between men and women, which may affect the pathophysiology of acute pancreatitis [29]. Our study results on the risk of acute pancreatitis by DPP-4i in female patients were similar to those of one observational study [5], but further well-designed studies are needed to confirm our results. The risk of pancreatitis by DPP-4i was lower than that by SU, regardless of age or DM microvascular complications. Lai et al. found an increased risk of pancreatitis in elderly (≥65 years) patients [5]. However, two other studies found that the risk of pancreatitis was not elevated in elderly patients, which was consistent with our findings [7, 28]. Two potential limitations of Lai et al.'s study were that their analysis was over a relatively short period of time (2 years, 2008 and 2009) and that they adjusted for relatively few confounding variables (alcohol use, hypertriglyceridemia, cholelithiasis, neoplasm, and the diabetes complications severity index). These limitations may have influenced their analysis.

Our study has several strengths. To our knowledge, this is the first retrospective observational study that has evaluated the risk of pancreatitis from DPP-4i after stratification according to the underlying CVD. In addition, we used a database that includes all claims data from primary clinics to tertiary teaching hospitals, representing > 99% of the South Korean population over 9 consecutive years. The mean follow-up of the matched patients was 627.6 days, which was longer than that of many other studies [5, 26–28], and thus may provide more compelling evidence. We also used a new user design with a 1-year washout period, which could reduce biases from retrospective nonrandomized comparative effectiveness studies [30]. Furthermore, we adjusted for 40 confounding variables.

Our research should be interpreted with caution, because our study also has limitations. Retrospective observational studies possess inherent limitations. We could not evaluate the details of each patient's medical history, including laboratory findings such as serum triglyceride, amylase, and lipase levels; alcohol use; tobacco use; or body mass index. However, we tried to compensate for this limitation as much as possible by adjusting for fibrate prescription and alcohol use according to the diagnostic codes for alcohol use, alcoholic cirrhosis, alcoholic gastritis, and alcohol-induced pancreatitis, and we set the outcome as hospitalization due to acute pancreatitis. Moreover, because this study is not a randomized controlled trial, residual confounding, such as duration of diabetes or socioeconomic status, may exist, as it could not be adjusted according to the diagnosis code. Further randomized control trials to estimate the risk of acute pancreatitis in DPP-4i use are needed to confirm our results in our study. Second, the severity of pancreatitis was not analyzed in our study. Pancreatitis severity is categorized according to results of abdominal computed tomography, laboratory tests, vital signs, and mental status, which were not included in the claims database used. In addition, there was no mortality data, rendering analysis of mortality due to pancreatitis impossible. The severity of SGLT-2i-induced pancreatitis should be estimated further in future randomized control trials.

## 5. Conclusions

In conclusion, our population-based observational cohort study showed that DPP-4i use was less likely to induce drug-induced pancreatitis compared with SU. This finding was not apparent in patients with CVD, but DPP-4i was not more likely to induce pancreatitis in these patients than SU. In addition, the lower tendency of DPP-4i to induce pancreatitis compared with SU was consistent regardless of age and DM microvascular complications, but this was not the case in female patients.

## Figures and Tables

**Table 1:** Baseline characteristics of matched pairs among all patients and among patients with and those without underlying cardiovascular disease.

	Total patients		Patients with underlying CVD		Patients without underlying CVD	
	SU	DPP-4i	SU	DPP-4i	SU	DPP-4i
*N*	13,091	13,091	3457	3457	9575	9575
Age (SD)	58.8 (12.9)	58.5 (12.5)	64.4 (11.6)	64.2 (11.4)	56.6 (12.8)	56.2 (12.5)
Sex (male, percent)	57.25	57.20	52.16	51.92	59.37	58.88
Hypertension	58.35	58.00	85.02	85.07	48.51	48.08
Dyslipidemia	58.99	59.62	74.34	74.49	53.67	54.39
Chronic kidney disease	5.22	5.32	8.53	8.71	4.13	4.47
Connective tissue disease	4.64	4.56	6.91	6.97	3.66	3.83
Cancer	6.55	6.66	8.16	8.42	5.83	5.97
Inflammatory bowel disease	0.17	0.15	0.23	0.20	0.13	0.14
Alcohol use^∗^	5.65	5.78	5.67	5.93	5.75	5.81
Tobacco use^∗^	0.05	0.07	0.17	0.14	0.02	0.02
Obesity^∗^	0.10	0.11	0.12	0.09	0.09	0.13
Hypoglycemia^∗^	0.53	0.50	1.01	0.95	0.33	0.36
Microvascular complications of diabetes						
Neuropathy	8.08	8.07	11.40	11.89	6.75	6.80
Nephropathy	4.19	4.13	6.07	6.36	3.56	3.78
Retinopathy	7.04	7.04	9.78	9.63	5.92	5.86
Disorder of hepatobiliary system						
Acute pancreatitis	0.60	0.60	0.84	0.81	0.52	0.50
Chronic pancreatitis	0.34	0.34	0.43	0.43	0.29	0.31
Gallstones	1.78	1.78	2.55	2.43	1.55	1.48
Liver cirrhosis	1.69	1.80	1.85	1.71	1.75	1.84
Primary biliary cirrhosis	0.02	0.02	0.00	0.00	0.00	0.01
Primary sclerosing cholangitis	0.13	0.15	0.20	0.23	0.14	0.11
Cardiovascular disease						
AMI	1.65	1.66	6.22	5.90		
Other ischemic heart disease	13.20	12.99	50.04	49.93		
Other heart disease	11.01	10.98	40.90	41.42		
Cerebral infarction	6.12	6.07	22.88	22.56		
Cerebrovascular event	7.79	7.72	28.90	28.52		
Peripheral artery disease	1.05	1.01	3.99	3.73		
Medication use						
Antidiabetic medicine						
Metformin	72.00	71.51	70.44	69.89	72.43	72.16
Alpha-glucosidase inhibitor	6.24	6.11	8.39	8.16	5.48	5.47
Thiazolidinediones	4.58	4.64	4.98	5.09	4.42	4.31
Meglitinide	2.47	2.35	3.48	3.37	2.01	1.99
SGLT2i	0.12	0.11	0.06	0.12	0.14	0.16
Insulin	11.19	11.31	17.50	17.53	8.77	9.21
Loop diuretics	6.57	6.76	15.30	14.93	3.38	3.84
Lipid-lowering agents						
Statin	33.89	34.49	49.67	50.10	28.31	28.81
Fibrate	4.77	4.93	5.87	5.67	4.30	4.72
Ezetimibe	1.67	1.75	3.15	3.38	1.09	1.44
PPI	13.75	13.61	18.28	18.14	11.99	12.32
ACEI/ARB	38.14	38.04	58.03	58.40	31.13	30.68
Pancreatobiliary procedure						
ERCP	0.11	0.11	0.09	0.12	0.11	0.13

Data presented as frequencies in percentage or means (standard deviation). ^∗^Confirmed by diagnosis code (International Classification of Diseases, 10th revision). The mean (standard deviation [SD]) standardized differences of all covariables were 0.46% (0.42%), 0.83% (0.48%), and 0.69% (0.71%) in all patients, those with underlying CVD, and those without underlying CVD, respectively. ACEI: angiotensin-converting enzyme inhibitor; AMI: acute myocardial infarction; ARB: angiotensin II receptor antagonists; CVD: cardiovascular disease; DPP-4i: dipeptidyl-peptidase IV inhibitor; ERCP: endoscopic retrograde cholangiopancreatography; *N*: number of patients; PPI: proton pump inhibitor; SD: standard deviation; SGLT2i: sodium-glucose cotransporter 2 inhibitor; SU: sulfonylurea.

**Table 2:** The risk of hospitalization for acute pancreatitis of DPP-4i new users as compared to SU new users.

	*N*	Events	HR	Lower CI	Upper CI	*P* value
Total patients	26,182	468	0.642	0.535	0.771	<0.001
Patients with underlying CVD	6914	150	0.727	0.527	1.003	0.052
Patients without underlying CVD	19,150	330	0.591	0.476	0.735	<0.001
Male	15,072	287	0.624	0.495	0.788	<0.001
Female	11,100	194	0.600	1.058	0.980	0.116
Patients aged ≥ 65 years	8718	207	0.717	0.545	0.943	0.017
Patients aged < 65 years	17,288	267	0.617	0.485	0.756	<0.001
Patients with DM microvascular complication	4326	91	0.542	0.357	0.822	0.004
Patients without DM microvascular complication	21,740	387	0.622	0.509	0.760	<0.001

CI: 95% confidence interval; CVD: cardiovascular disease; DM: diabetes mellitus; DPP-4i: dipeptidyl-peptidase IV inhibitor; HR: hazard ratio; *N*: number of patients; SU: sulfonylurea.

## Supplementary Materials

Supplementary Materials Supplementary Table 1: baseline characteristics of matched pairs in male and female patients. Data presented as frequencies in percentage or means (standard deviation). ^∗^Confirmed by diagnosis code (International Classification of Diseases, 10th revision). The mean (SD) standardized differences of all covariables were 0.64% (0.54%) and 0.82% (0.68%) in male and female patients, respectively. ACEI: angiotensin-converting enzyme inhibitor; AMI: acute myocardial infarction; ARB: angiotensin II receptor antagonists; CVD: cardiovascular disease; DPP-4i: dipeptidyl-peptidase IV inhibitor; ERCP: endoscopic retrograde cholangiopancreatography; N: number of patients; PPI: proton pump inhibitor; SD: standard deviation; SGLT2i: sodium-glucose cotransporter 2 inhibitor; SU: sulfonylurea. Supplementary Table 2: baseline characteristics of matched pairs in young and elderly patients. Data presented as frequencies in percentage or means (standard deviation). ^∗^Confirmed by diagnosis code (International Classification of Diseases, 10th revision). The mean (SD) standardized differences of all covariables were 0.84% (0.51%) and 0.57% (0.57%) in elderly and young patients, respectively. ACEI: angiotensin-converting enzyme inhibitor; AMI: acute myocardial infarction; ARB: angiotensin II receptor antagonists; CVD: cardiovascular disease; DPP-4i: dipeptidyl-peptidase IV inhibitor; ERCP: endoscopic retrograde cholangiopancreatography; N: number of patients; PPI: proton pump inhibitor; SD: standard deviation; SGLT2i: sodium-glucose cotransporter 2 inhibitor; SU: sulfonylurea. Supplementary Table 3: baseline characteristics of matched pairs in patients with or without underlying diabetes mellitus microvascular complication. Data presented as frequencies in percentage or means (standard deviation). ^∗^Confirmed by diagnosis code (International Classification of Diseases, 10th revision). The mean (SD) standardized differences of all covariables were 1.08% (0.98%) and 0.52% (0.58%) in patients with or without DM microvascular complication, respectively. ACEI: angiotensin-converting enzyme inhibitor; AMI: acute myocardial infarction; ARB: angiotensin II receptor antagonists; CVD: cardiovascular disease; DM: diabetes mellitus; DPP-4i: dipeptidyl-peptidase IV inhibitor; ERCP: endoscopic retrograde cholangiopancreatography; N: number of patients; PPI: proton pump inhibitor; SD: standard deviation; SGLT2i: sodium-glucose cotransporter 2 inhibitor; SU: sulfonylurea.
