# Supplementary material for: Dipeptidyl Peptidase-4 Inhibitors and the Risk of Pancreatitis in Patients with Type 2 Diabetes Mellitus: A Population-Based Cohort Study
Source: J Diabetes Res. 2018 Apr 10;2018:5246976. doi: 10.1155/2018/5246976 (PMC5914097; doi:10.1155/2018/5246976)
Supplement: Supplementary Materials — Supplementary Table 1: baseline characteristics of matched pairs in male and female patients. Data presented as frequencies in percentage or means (standard deviation). ∗Confirmed by diagnosis code (International Classification of Diseases, 10th revision). The mean (SD) standardized differences of all covariables were 0.64% (0.54%) and 0.82% (0.68%) in male and female patients, respectively. ACEI: angiotensin-converting enzyme inhibitor; AMI: acute myocardial infarction; ARB: angiotensin II receptor antagonists; CVD: cardiovascular disease; DPP-4i: dipeptidyl-peptidase IV inhibitor; ERCP: endoscopic retrograde cholangiopancreatography; N: number of patients; PPI: proton pump inhibitor; SD: standard deviation; SGLT2i: sodium-glucose cotransporter 2 inhibitor; SU: sulfonylurea. Supplementary Table 2: baseline characteristics of matched pairs in young and elderly patients. Data presented as frequencies in percentage or means (standard deviation). ∗Confirmed by diagnosis code (International Classification of Diseases, 10th revision). The mean (SD) standardized differences of all covariables were 0.84% (0.51%) and 0.57% (0.57%) in elderly and young patients, respectively. ACEI: angiotensin-converting enzyme inhibitor; AMI: acute myocardial infarction; ARB: angiotensin II receptor antagonists; CVD: cardiovascular disease; DPP-4i: dipeptidyl-peptidase IV inhibitor; ERCP: endoscopic retrograde cholangiopancreatography; N: number of patients; PPI: proton pump inhibitor; SD: standard deviation; SGLT2i: sodium-glucose cotransporter 2 inhibitor; SU: sulfonylurea. Supplementary Table 3: baseline characteristics of matched pairs in patients with or without underlying diabetes mellitus microvascular complication. Data presented as frequencies in percentage or means (standard deviation). ∗Confirmed by diagnosis code (International Classification of Diseases, 10th revision). The mean (SD) standardized differences of all covariables were 1.08% (0.98%) and 0.52% (0.58%) in patients wi [file 5246976.f1.docx]

**Supplementary Materials**

**Dipeptidyl peptidase-4 inhibitors and the risk of pancreatitis in patients**

**with type 2 diabetes mellitus: A population-based cohort study**

**Supplementary Table 1. Baseline characteristics of matched pairs in male and female patients**

|  | Male | | Female | |
| --- | --- | --- | --- | --- |
|  | SU | DPP-4i | SU | DPP-4i |
| N | 7536 | 7536 | 5523 | 5523 |
| Age (SD) | 56.0 (12.1) | 56.1 (12.1) | 62.1 (12.7) | 61.8 (12.5) |
| Sex (Male, percent) | 100.00 | 100.00 | 0.00 | 0.00 |
| Hypertension | 53.36 | 53.69 | 65.04 | 64.17 |
| Dyslipidemia | 55.76 | 56.41 | 63.62 | 63.81 |
| Chronic kidney disease | 5.18 | 5.29 | 5.12 | 5.49 |
| Connective tissue disease | 2.85 | 2.85 | 6.70 | 6.70 |
| Cancer | 6.82 | 6.86 | 6.19 | 6.08 |
| Inflammatory Bowel disease | 0.15 | 0.16 | 0.11 | 0.20 |
| Alcohol use* | 8.15 | 8.32 | 2.06 | 2.17 |
| Tobacco use* | 0.07 | 0.08 | 0.00 | 0.02 |
| Obesity* | 0.03 | 0.03 | 0.18 | 0.18 |
| Hypoglycemia* | 0.46 | 0.45 | 0.62 | 0.58 |
| Microvascular complications of diabetes |  |  |  |  |
| Neuropathy | 7.18 | 7.46 | 8.98 | 8.76 |
| Nephropathy | 4.18 | 4.22 | 4.06 | 4.18 |
| Retinopathy | 6.82 | 6.71 | 7.37 | 7.55 |
| Disorder of hepatobiliary system |  |  |  |  |
| Acute pancreatitis | 0.66 | 0.58 | 0.47 | 0.51 |
| Chronic pancreatitis | 0.38 | 0.33 | 0.25 | 0.31 |
| Gallstones | 1.75 | 1.76 | 1.68 | 1.94 |
| Liver cirrhosis | 2.14 | 2.45 | 0.94 | 1.00 |
| Primary biliary cirrhosis | 0.00 | 0.01 | 0.02 | 0.02 |
| Primary sclerosing cholangitis | 0.23 | 0.19 | 0.07 | 0.09 |
| Cardiovascular disease |  |  |  |  |
| AMI | 1.90 | 1.88 | 1.12 | 1.27 |
| Other ischemic heart disease | 12.53 | 12.58 | 14.47 | 14.52 |
| Other heart disease | 8.92 | 9.18 | 13.36 | 13.40 |
| Cerebral infarction | 5.51 | 5.63 | 6.64 | 6.95 |
| Cerebrovascular event | 7.01 | 7.15 | 8.58 | 8.85 |
| Peripheral artery disease | 0.98 | 0.90 | 1.16 | 1.09 |
| Medication use |  |  |  |  |
| Anti-diabetic medicine |  |  |  |  |
| Metformin | 72.23 | 72.11 | 71.41 | 70.89 |
| Alpha-glucosidase inhibitor | 6.08 | 6.09 | 6.48 | 6.45 |
| Thiazolidinediones | 4.76 | 4.55 | 4.25 | 4.51 |
| Meglitinide | 2.23 | 2.26 | 2.59 | 2.70 |
| SGLT2i | 0.13 | 0.15 | 0.11 | 0.11 |
| Insulin | 11.23 | 11.28 | 11.23 | 11.75 |
| Loop diuretics | 5.32 | 5.61 | 8.18 | 8.69 |
| Lipid-lowering agents |  |  |  |  |
| Statin | 28.82 | 29.11 | 40.54 | 41.03 |
| Fibrate | 5.72 | 5.67 | 3.57 | 3.57 |
| Ezetimibe | 1.41 | 1.63 | 1.97 | 2.08 |
| PPI | 12.54 | 12.95 | 15.28 | 15.25 |
| ACEI/ARB | 36.61 | 36.58 | 40.59 | 40.32 |
| Pancreatobiliary procedure |  |  |  |  |
| ERCP | 0.15 | 0.13 | 0.07 | 0.13 |

Data presented as frequencies in percentage or means (standard deviation).

*Confirmed by diagnosis code (International Classification of Diseases, 10^th^ revision)

The mean (SD) standardized differences of all covariables were 0.64% (0.54%), 0.82% (0.68%) in male and female patients, respectively.

ACEI, angiotensin-converting-enzyme inhibitor; AMI, acute myocardial infarction; ARB, angiotensin II receptor antagonists; CVD, cardiovascular disease; DPP-4i, dipeptidyl-peptidase IV inhibitor; ERCP, Endoscopic Retrograde Cholangio-Pancreatography; N, number of patients; PPI, proton pump inhibitor; SD, standard deviation; SGLT2i, sodium-glucose co-transporter 2 inhibitor; SU, sulfonylurea.

**Supplementary Table 2. Baseline characteristics of matched pairs in young and elderly patients**

|  | Elderly (≥ 65 years) | | Young (< 65 years) | |
| --- | --- | --- | --- | --- |
|  | SU | DPP-4i | SU | DPP-4i |
| N | 4359 | 4359 | 8644 | 8644 |
| Age (SD) | 72.5 (5.7) | 72.6 (5.7) | 51.3 (8.8) | 51.2 (8.5) |
| Sex (Male, percent) | 43.34 | 43.63 | 64.98 | 64.82 |
| Hypertension | 76.21 | 76.35 | 49.17 | 48.87 |
| Dyslipidemia | 61.37 | 61.99 | 57.76 | 58.87 |
| Chronic kidney disease | 6.31 | 6.22 | 4.88 | 5.21 |
| Connective tissue disease | 6.40 | 6.65 | 3.64 | 3.75 |
| Cancer | 9.50 | 9.27 | 5.29 | 5.18 |
| Inflammatory Bowel disease | 0.16 | 0.21 | 0.14 | 0.17 |
| Alcohol use* | 3.62 | 3.46 | 7.00 | 7.23 |
| Tobacco use* | 0.02 | 0.05 | 0.07 | 0.08 |
| Obesity* | 0.09 | 0.05 | 0.10 | 0.13 |
| Hypoglycemia* | 0.85 | 0.78 | 0.39 | 0.31 |
| Microvascular complications of diabetes |  |  |  |  |
| Neuropathy | 9.91 | 10.16 | 7.02 | 6.84 |
| Nephropathy | 4.47 | 4.36 | 4.11 | 4.42 |
| Retinopathy | 8.24 | 8.42 | 6.36 | 6.32 |
| Disorder of hepatobiliary system |  |  |  |  |
| Acute pancreatitis | 0.50 | 0.48 | 0.66 | 0.65 |
| Chronic pancreatitis | 0.25 | 0.21 | 0.38 | 0.37 |
| Gallstones | 2.20 | 2.16 | 1.67 | 1.58 |
| Liver cirrhosis | 1.54 | 1.47 | 1.80 | 2.09 |
| Primary biliary cirrhosis | 0.00 | 0.02 | 0.01 | 0.01 |
| Primary sclerosing cholangitis | 0.25 | 0.21 | 0.10 | 0.09 |
| Cardiovascular disease |  |  |  |  |
| AMI | 2.34 | 2.34 | 1.27 | 1.23 |
| Other ischemic heart disease | 20.95 | 21.38 | 9.35 | 9.31 |
| Other heart disease | 17.64 | 17.37 | 7.73 | 7.66 |
| Cerebral infarction | 11.65 | 11.65 | 3.16 | 3.16 |
| Cerebrovascular event | 14.04 | 14.34 | 4.40 | 4.35 |
| Peripheral artery disease | 1.51 | 1.56 | 0.79 | 0.79 |
| Medication use |  |  |  |  |
| Anti-diabetic medicine |  |  |  |  |
| Metformin | 70.98 | 71.32 | 71.89 | 72.08 |
| Alpha-glucosidase inhibitor | 7.34 | 7.41 | 5.54 | 5.59 |
| Thiazolidinediones | 4.43 | 4.50 | 4.62 | 4.73 |
| Meglitinide | 3.42 | 3.40 | 1.94 | 1.96 |
| SGLT2i | 0.05 | 0.07 | 0.16 | 0.17 |
| Insulin | 13.54 | 13.86 | 10.06 | 10.19 |
| Loop diuretics | 11.33 | 11.81 | 4.37 | 4.45 |
| Lipid-lowering agents |  |  |  |  |
| Statin | 37.99 | 38.43 | 31.76 | 32.51 |
| Fibrate | 3.17 | 3.28 | 5.68 | 5.65 |
| Ezetimibe | 1.61 | 1.88 | 1.61 | 1.62 |
| PPI | 16.22 | 16.52 | 12.38 | 12.48 |
| ACEI/ARB | 47.67 | 47.81 | 33.46 | 33.24 |
| Pancreatobiliary procedure |  |  |  |  |
| ERCP | 0.18 | 0.14 | 0.07 | 0.08 |

Data presented as frequencies in percentage or means (standard deviation).

*Confirmed by diagnosis code (International Classification of Diseases, 10^th^ revision)

The mean (SD) standardized differences of all covariables were 0.84% (0.51%), 0.57% (0.57%) in elderly and young patients, respectively.

ACEI, angiotensin-converting-enzyme inhibitor; AMI, acute myocardial infarction; ARB, angiotensin II receptor antagonists; CVD, cardiovascular disease; DPP-4i, dipeptidyl-peptidase IV inhibitor; ERCP, Endoscopic Retrograde Cholangio-Pancreatography; N, number of patients; PPI, proton pump inhibitor; SD, standard deviation; SGLT2i, sodium-glucose co-transporter 2 inhibitor; SU, sulfonylurea.

**Supplementary Table 3. Baseline characteristics of matched pairs in patients with or without underlying diabetes mellitus microvascular complication**

|  | Patients with underlying DM microvascular complication | | Patients without underlying DM microvascular complication | |
| --- | --- | --- | --- | --- |
|  | SU | DPP-4i | SU | DPP-4i |
| N | 2163 | 2163 | 10870 | 10870 |
| Age (SD) | 60.3 (12.7) | 60.0 (12.3) | 58.4 (13.0) | 58.2 (12.5) |
| Sex (Male, percent) | 54.88 | 54.88 | 57.81 | 57.35 |
| Hypertension | 64.68 | 64.17 | 57.07 | 56.96 |
| Dyslipidemia | 68.15 | 68.24 | 57.09 | 57.19 |
| Chronic kidney disease | 26.95 | 27.65 | 0.90 | 0.88 |
| Connective tissue disease | 6.15 | 6.24 | 4.20 | 4.17 |
| Cancer | 7.63 | 7.35 | 6.24 | 6.25 |
| Inflammatory Bowel disease | 0.14 | 0.23 | 0.16 | 0.19 |
| Alcohol use* | 5.22 | 5.27 | 5.69 | 5.69 |
| Tobacco use* | 0.00 | 0.05 | 0.04 | 0.05 |
| Obesity* | 0.05 | 0.14 | 0.11 | 0.11 |
| Hypoglycemia* | 1.20 | 1.11 | 0.40 | 0.38 |
| Microvascular complications of diabetes |  |  |  |  |
| Neuropathy | 49.01 | 48.17 |  |  |
| Nephropathy | 24.97 | 25.47 |  |  |
| Retinopathy | 41.75 | 41.56 |  |  |
| Disorder of hepatobiliary system |  |  |  |  |
| Acute pancreatitis | 0.79 | 0.83 | 0.56 | 0.58 |
| Chronic pancreatitis | 0.83 | 0.55 | 0.26 | 0.26 |
| Gallstones | 2.45 | 2.64 | 1.70 | 1.67 |
| Liver cirrhosis | 2.17 | 2.27 | 1.55 | 1.70 |
| Primary biliary cirrhosis | 0.00 | 0.00 | 0.02 | 0.01 |
| Primary sclerosing cholangitis | 0.14 | 0.14 | 0.16 | 0.16 |
| Cardiovascular disease |  |  |  |  |
| AMI | 2.54 | 2.54 | 1.44 | 1.42 |
| Other ischemic heart disease | 19.74 | 18.77 | 11.97 | 12.21 |
| Other heart disease | 15.16 | 15.77 | 10.06 | 10.17 |
| Cerebral infarction | 9.06 | 8.97 | 5.54 | 5.48 |
| Cerebrovascular event | 11.14 | 11.10 | 5.54 | 5.48 |
| Peripheral artery disease | 1.71 | 1.71 | 7.12 | 7.07 |
| Medication use |  |  |  |  |
| Anti-diabetic medicine |  |  |  |  |
| Metformin | 75.91 | 75.54 | 71.21 | 70.79 |
| Alpha-glucosidase inhibitor | 10.82 | 10.82 | 5.33 | 5.18 |
| Thiazolidinediones | 7.35 | 7.12 | 3.96 | 4.14 |
| Meglitinide | 6.29 | 6.33 | 1.57 | 1.62 |
| SGLT2i | 0.23 | 0.23 | 0.10 | 0.13 |
| Insulin | 23.44 | 22.52 | 8.77 | 8.76 |
| Loop diuretics | 9.94 | 10.12 | 5.87 | 6.30 |
| Lipid-lowering agents |  |  |  |  |
| Statin | 40.22 | 39.57 | 32.58 | 32.91 |
| Fibrate | 5.69 | 6.10 | 4.57 | 4.63 |
| Ezetimibe | 2.31 | 2.40 | 1.52 | 1.89 |
| PPI | 15.16 | 14.10 | 13.43 | 13.68 |
| ACEI/ARB | 45.26 | 44.52 | 36.87 | 36.98 |
| Pancreatobiliary procedure |  |  |  |  |
| ERCP | 0.09 | 0.14 | 0.14 | 0.13 |

Data presented as frequencies in percentage or means (standard deviation).

*Confirmed by diagnosis code (International Classification of Diseases, 10^th^ revision)

The mean (SD) standardized differences of all covariables were 1.08% (0.98%), 0.52% (0.58%) in patients with or without DM microvascular complication, respectively.

ACEI, angiotensin-converting-enzyme inhibitor; AMI, acute myocardial infarction; ARB, angiotensin II receptor antagonists; CVD, cardiovascular disease; DM, diabetes mellitus; DPP-4i, dipeptidyl-peptidase IV inhibitor; ERCP, Endoscopic Retrograde Cholangio-Pancreatography; N, number of patients; PPI, proton pump inhibitor; SD, standard deviation; SGLT2i, sodium-glucose co-transporter 2 inhibitor; SU, sulfonylurea.
